# Using Social Marketing to Reduce Intention of Cesarean Section in Iranian Women

**DOI:** 10.1155/2021/3920126

**Published:** 2021-01-27

**Authors:** Mostafa Maleki, Ali Mousavizadeh, Saadat Parhizkar, Mohsen Shams

**Affiliations:** ^1^Ph.D. Candidate in Health Education and Promotion, Department of Health Education and Promotion, School of Health, Tehran University of Medical Sciences, Tehran, Iran; ^2^Ph.D. in Epidemiology, Assistant Professor, Social Determinants of Health Research Center, Yasuj University of Medical Sciences, Yasuj, Iran; ^3^Ph.D. in Reproductive Health, Associate Professor, Social Determinants of Health Research Center, Yasuj University of Medical Sciences, Yasuj, Iran; ^4^Ph.D. in Health Education, Associate Professor, Social Determinants of Health Research Center, Yasuj University of Medical Sciences, Yasuj, Iran

## Abstract

**Introduction:**

In Iran, the rate of cesarean section is three times more than the acceptable rate considered by the World Health Organization. This study aimed at reducing the selection of cesarean section by primigravida through an intervention based on social marketing in Boyer-Ahmad County, Iran, 2015.

**Methods:**

In this field trial, 39 of primigravida women were identified and selected as a target group. Formative research (a quantitative survey and a qualitative study) was done to achieve the social marketing mix. The tailored intervention was developed based on the findings of formative research. The intervention was implemented for one month for pregnant women who had cesarean section intention. Their intention for the cesarean section was studied again one month after the implementation of the intervention. The effectiveness of the intervention was evaluated by the proportion test.

**Results:**

The average age of the women was 25.82. All of the women 38.5 percent had a diploma degree and lower than and 61.5 percent had a university degree. Before the intervention, 39 women intended to do Cesarean. The intention of 30 pregnant women was changed significantly one month after the intervention.

**Conclusion:**

The study showed the effectiveness of an intervention based on consumer-oriented social marketing theory and could be used to reduce Cesarean intention. More studies about related factors of vaginal delivery selection especially from behavioral intention up to behavior are suggested.

## 1. Introduction

The World Health Organization (WHO) in 1985 reported that the rate of the cesarean section should not exceed 10–15 percent anywhere in the world [1]. The WHO in 2015 has also emphasized that if the cesarean sections are performed due to medically indicated reasons, it could be effective in saving maternal and infant lives, while the cesarean section rates over 10% have no association with the decreased maternal and newborn mortality [2]. However, in Iran, the cesarean section rate has risen from 35% in 2000 up to 47.9% in 2009 [3].

The main goal of the cesarean section is to reduce pregnant women's and infants' complications, mortality, and morbidity. This goal has been largely achieved over the last few decades, but in recent years, this goal has been challenged by a significant increase in delivery via cesarean section. Cesarean delivery without medical indication can develop significant maternal and infant complications [4, 5]. High rates of the cesarean section could result in maternal complications, such as respiratory infections, pulmonary embolism and bleeding, neonatal respiratory distress syndrome, and longer hospitalization time of infants in neonatal intensive care units [6, 7]. The maternal mortality rate in cesarean delivery is 2 to 3 times higher than the normal delivery [8]. The increased complications resulted from the high prevalence of the cesarean section have challenged the initial goal of cesarean. Therefore, effective interventions to reduce cesarean delivery should be considered as a priority of public health.

Maternal demand is a significant driver of the overall cesarean section rate in Iran [9–12]. Also, first pregnancy, prenatal care, and delivery type in first pregnancy have a great influence on their experience and feelings of pregnancy and delivery; all of these can determine the type of next childbirth [13]. Previous studies have indicated that the high rates of cesarean section among primigravida and repeated cesarean section are of the main reasons for high cesarean section rates [4, 14]. Therefore, interventions reducing cesarean delivery in the first pregnancy can significantly reduce the cesarean section rate.

Viewpoints of the target group in most of the interventions for reducing the cesarean section in Iran are less considered [15–17]. However, the needs of the target group in social marketing are collected through appropriate research methods and are considered in the design of an intervention to increase the benefits of behavior, decrease its barriers, or provide incentives for the desired behavior. This approach is effective for those who are in a competitive position and tended to perform competitive behavior. Cutler and Roberto in 1989 defined social marketing as a planning process promoting voluntary behavior in the audience by presenting their desired benefits, reducing the barriers, and motivating them to promote individual and social welfare [18]. The use of social marketing requires the selection of an appropriate operational framework such as Social Marketing Assessment and Response Tool (SMART), which is developed by Neiger in seven phases including preliminary planning, consumer analysis, market analysis, and channel analysis, development of interventions, materials, and pretest, implementation as well as evaluation [19]. Given the effectiveness of social marketing in promoting public health, the present study aimed to investigate the effect of intervention based on social marketing in reducing the intention of elective cesarean section among women with first pregnancy in Boyer-Ahmad County, Iran.

## 2. Methods

This study was aimed to reduce the selection of the cesarean section by primigravida. For this purpose, we developed and conducted formative research (qualitative and quantitative studies) and the intervention phase. Report of formative research was published in another journal, but in summary, 37 pregnant women in their first pregnancy participated in a focus group discussion and 12 individuals from health care providers were interviewed trough in-depth interviews in the qualitative study. In the quantitative survey, structures of the theory of planned behavior were determined for 157 pregnant women in their first pregnancy using a standard questionnaire [9].

The intervention phase is reported in this paper. The current study was a field trial based on the SMART model. Approval for the research was obtained from Yasuj University of Medical Sciences Ethics Committee (I.R.REC.1394.52) before the study commenced. At the beginning of each stage of the study, the aim of the study was explained to the participants and their informed consent was obtained.

In the present study, the pregnant women in their first pregnancy were assigned to a specific target group. The main aim of this study was to change the intention of pregnant women from cesarean delivery to normal delivery; hereby, the duration of the design and implementation of the intervention was calculated to be four months; accordingly, all pregnant women in their first pregnancy at the gestational age of three and four months were enrolled in the study. According to the analysis of the developmental research, the intervention program included education courses, brief interventions through suitable channels for pregnant women (physician and midwife) in health centers, childbirth classes, concerns on barriers to participate in education courses for reducing the intervention rate, and transfer and promote the messages of the program through free telephone counseling by individuals influencing pregnant women. The number of pregnant women intended to cesarean delivery was also determined. The designed intervention was applied at the same time for all pregnant women intended to the cesarean section for one month. Education courses were held at the determined time for pregnant women (10-11 : 30) so that most of them could participate in classes. Overall, eight educational sessions were held. Based on the findings of the developmental research, the content of these educational sessions was to correct the attitude and subjective norms of pregnant women regarding the choice of delivery type. Freephone counseling was used to record the messages of the education courses and transfer them to women who did not attend the classes. For this purpose, a counseling card was prepared and distributed among the target group and asked them whether they or those who influence the choice of delivery (wife, mother, sister, and mother-in-law) to call the given phone number to receive free phone counseling. According to the opinion of women in the target group, the time for telephone counseling was considered to be 9-11 am and 3-5 pm. Two senior midwifery students were selected as telephone counselors after receiving pieces of training on how to give telephone counseling for pregnant women.

**Figure 1:**
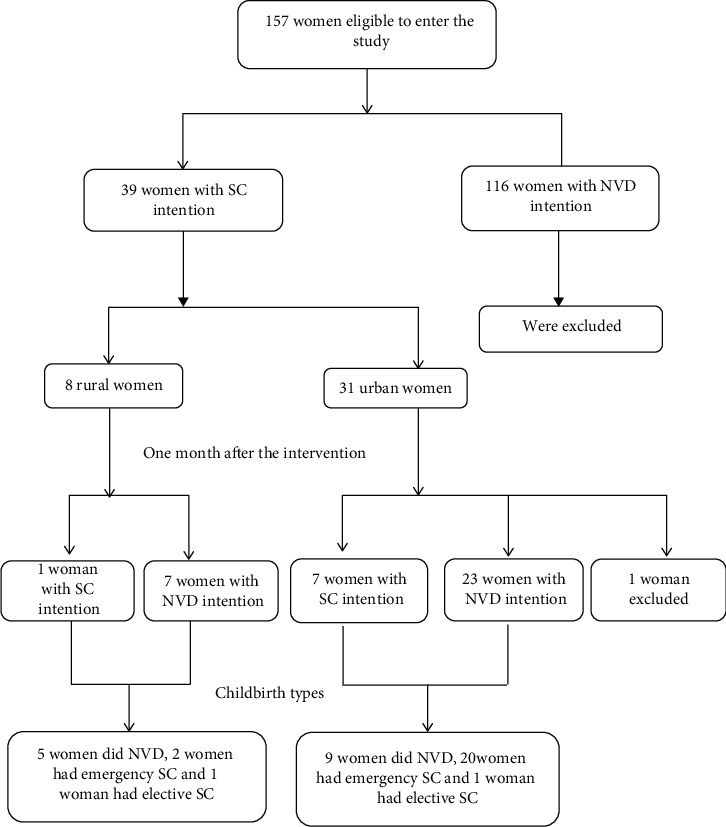
Study flowchart.

According to the findings of the formative research, the physicians and midwives of health centers simultaneously with the education courses and free telephone counseling conducted the brief intervention to reduce the fear of normal delivery in pregnant women at their first pregnancy. The brief intervention guideline was developed based on the findings of the developmental research and presented to the physicians and midwives. To this end, the physicians and midwives of health centers were requested to ask all of the referred pregnant women at their first pregnancy about the chosen delivery type, and if they had chosen the cesarean section, they would have informed this message: “contrary to popular belief, cesarean section is not painless, and severe and prolonged pain begins after delivery.” Due to the large dispersion of the rural pregnant women planning to have a cesarean section, education courses were not held for them, but they received telephone counseling and a brief intervention. After about four weeks, the intention of pregnant women's choice of delivery type was determined again through the telephone survey. Since the intervention was implemented for those pregnant women who had planned to undergo a cesarean section, the comparative group was not considered for this group, and the researchers considered a reduction rate of 60% in the cesarean section as a comparative criterion for assessing the effectiveness of the intervention. The ratio test was used to evaluate the effectiveness of the intervention. The study flowchart represents various stages of the study (Figure 1). One hundred counseling sessions were performed over one month. The mean time spent on each phone consultation was five minutes. The shortest and longest telephone counseling times were one and six minutes, respectively.

## 3. Results

A total number of 198 eligible pregnant women were identified. Of these, 157 pregnant women participated in the formative study, 39 of them had decided for the cesarean section. Because the aim of the study was reducing the intention of elective cesarean section, therefore, these 39 women were selected as a target population of the intervention phase. The mean and standard deviation age of the target population was 25.82 ± 3.91 years and the minimum and maximum age of them were 18 and 34 years. Of these, 38.5% of the women had high school or lower diplomas and 61.5% had academic education. As well, 79.5% of the women planned to perform the cesarean section were living in cities and 20.5% were living in the villages. Further information on this group of women is presented in Table 1.

### 3.1. Findings of Formative Research

In the results of the qualitative study, various types of interventions were proposed by participants to reduce cesarean section rates included holding education courses for pregnant women by providing educational content based on their needs and desires, telephone counseling for the pregnant women and their husbands, the brief intervention by the physicians and midwives of health centers, and provision of a pleasant environment for the women referring to the maternity ward. The results of the quantitative survey showed that the intention of the cesarean section was 44.3% (*n* = 31) and 9.4% (*n* = 8) among urban- and rural-dwelling pregnant women, respectively (*P* < 0.001). Regardless of their location, the intention of the cesarean section was 25.2% (*n* = 39).

According to the quantitative data analysis, the strongest predictors of the intention of the delivery type were found to be the subjective norms (OR = 2.413, *P* = 0.021, CI = 1.22 − 2.51) and attitude to normal delivery (OR = 1.754, *P* = 0.002, CI = 1.14 − 5.08), which was the basis of content production. It was also indicated that the providers of health care services in health centers (physician and midwife), pregnant women, and their husbands had the most impact on selecting the delivery type [9].

### 3.2. The Results of Intervention Assessment

The posttest results showed that the designed intervention succeeded to change the intention of 78.9% (30 out of 38) of all pregnant women who preferred to perform the cesarean section before intervention (*P* = 0.01). Concerning the residence, 76.7% (23 out of 30 people) of urban-dwelling women (*P* = 0.04) and 87.5% (7 out of 8 people) of rural-dwelling women who tended to undergo the cesarean section before the intervention have changed their intention (Table 2). Of these, 36.84% (*n* = 14) performed normal delivery, 57.8% (*n* = 22) experienced emergency cesarean section, and 5.2% (*n* = 2) underwent elective cesarean section. The intention of the target group before and after the intervention and the delivery type are given in the flowchart presented in the methodology section of the study.

## 4. Discussion

Pregnant women with their first pregnancy are considered the most important groups for interventions to reduce cesarean section rates. Correcting the intention of the delivery type among this group can have a significant effect on the reduction of cesarean section rates. The present study aimed to investigate the effect of the intervention based on the social marketing model in decreasing the intention toward selecting a cesarean section among pregnant women at their first pregnancy in Boyer-Ahmad County, Iran. The social marketers believe that if an idea or behavior presented to a target group is tailored to their needs and aspirations, or increases the benefits of adopting the desired idea or behavior and reduces its barriers compared with competitor behavior or ideas, the ability to accept that idea or behavior could be improved in the target group [20]. In the present study, a combination obtained from the qualitative research and quantitative survey and their findings were used to design an intervention tailored to the needs and aspirations of pregnant women at their first pregnancy as the specific target group. The educational sessions and free telephone counseling provided the conditions for pregnant women who could easily talk about their concerns, fears, stresses, and anxieties and, hereby, overcome their mental barriers to choose the natural childbirth.

In the current study, the social marketing-based interventions succeeded in significantly changed the intention of pregnant women from cesarean section to normal delivery. However, only 36.84% (14 out of 38 people) had normal delivery, 57.8% (22 out of 38 people) underwent emergency cesarean section, and 5.2% (2 out of 38 people) had an elective cesarean section. In the study of Tofighinia et al., despite that the designed intervention could reduce 80.6% of the intention of the cesarean section, only 56.7% of them experienced normal delivery [21]. The educational intervention designed based on the rational choice theory also succeeded to significantly decrease the intention of performing cesarean delivery among women in the intervention group, but only 21 out of 45 people had natural deliveries, which was not significant compared to the control group [15]. In another study, the target group was pregnant women at their first pregnancy; it was found that, even though 60% of the pregnant women intended to have a normal delivery, 50% had eventually undergone cesarean delivery [22]. Evaluating conditions of the hospitals and physicians in addition to pregnant women and assessing the root causes of the cesarean section can help to better understand this issue. In a study on six controlled trials, only 2 interventions with relaxation training programs for nurses to approach the pregnant mothers and with a childbirth preparation educational session have been effective in reducing cesarean section rates. Also, reviewing 10 controlled trials emphasizing physicians indicated that only 3 trials have been effectively reduced cesarean delivery [15]. Since various behavioral and nonbehavioral factors result in the tendency of pregnant women to perform a cesarean section, it seems that single-dimensional interventions have a limited ability to change the behavior of pregnant women. Interventions that consider all or some of these factors that have the greatest impact on changing the behavior of the target group can be successful in reducing the intention of pregnant women and changing their behavior.

In this regard, the comprehensive patient safety program in the United States could reduce the cesarean section rate from 41.6% in 2004 down to 32.7% in 2012 [23]. This program included educational interventions, correcting the cesarean indications, and the provision of services for pregnant women. Runmei et al. also concluded that multivariate educational interventions for pregnant women, training service providers, and the performance audit of obstetricians are effective for reducing cesarean section rate [24].

One of the considerable points in the present study is the high percentage of emergency cesarean section rate (57.8%). The cause of this number of emergency cesarean section is questionable and requires further investigations. Perhaps in the Iran context, obstetricians, who benefit from cesarean delivery, perform cesarean delivery due to fake indications. Some service providers mentioned to this issue within the individual interviews. A performance audit of obstetricians and surgeons and further studies on factors related to the choice of delivery method especially from the intent toward perform a behavior until its onset can help to better understand this issue. Our findings may not be generalized to other pregnant women, because primigravida women were the only target audience of the present study.

## 5. Conclusion

The social marketing provides a scientific and effective framework for designing, implementing, and assessing the interventions for reducing the intention of performing a cesarean delivery. It is suggested to be conducted further studies on the factors affecting the choice of the delivery type especially from the intent to perform a behavior until its onset.

## Figures and Tables

**Table 1:** Demographic information of pregnant women with the intention of cesarean section and normal vaginal delivery.

Demographic variables		Women with cesarean section		Women with normal vaginal delivery	
		Number	Percentage	Number	Number
The education level of pregnant women	Diploma and under diploma	15	38.5	75	64.65
	Academic	24	61.5	41	35.35
The education level of husband	Diploma and under diploma	16	41	66	56.5
	Academic	23	59	50	43.5
Age groups	Less than 20 years	4	10.3	24	20.5
	21–30 years	30	76.9	77	66.8
	More than 31 years	5	12.8	14	12.7
The job of pregnant women	Housewife	29	74.4	105	90
	Employed	7	17.9	5	4.5
	Student	2	5.1	6	5.5
Job of husband	Employed	18	46.2	22	19.8
	Self-employment	20	51.3	80	70.5
	Unemployed	1	2.6	11	9.7
Place of residence	Rural	31	79.5	77	33.5
	Urban	8	20.5	39	66.5
Status of pregnancy	Planned	34	87.2	100	88.5
	Unplanned	5	12.8	13	11.5

**Table 2:** Change of intention among primigravida women based on ratio test.

Target group	Women who changed the intention	Total	Percentage of change	Sig.
All women	30	38	.79	0.01^∗^
Urban women	23	30	.77	0.04^∗^
Rural women	7	8	.87	0.1

^∗^Based on the ratio test with a cut of point of 0.6.

## Data Availability

The datasets used and/or analyzed during the current study are available from the corresponding author on reasonable request.
